# Hyperprolactinemia and Hypopituitarism in Acromegaly and Effect of Pituitary Surgery: Long-Term Follow-up on 529 Patients

**DOI:** 10.3389/fendo.2021.807054

**Published:** 2022-01-26

**Authors:** Xiaopeng Guo, Ruopeng Zhang, Duoxing Zhang, Zihao Wang, Lu Gao, Yong Yao, Kan Deng, Xinjie Bao, Ming Feng, Zhiqin Xu, Yi Yang, Wei Lian, Renzhi Wang, Wenbin Ma, Bing Xing

**Affiliations:** ^1^ Department of Neurosurgery, Peking Union Medical College Hospital, Chinese Academy of Medical Sciences and Peking Union Medical College, Beijing, China; ^2^ Key Laboratory of Endocrinology of Ministry of Health, Peking Union Medical College Hospital, Chinese Academy of Medical Sciences and Peking Union Medical College, Beijing, China; ^3^ China Pituitary Disease Registry Center, Beijing, China; ^4^ China Pituitary Adenoma Specialist Council, Beijing, China; ^5^ Peking Union Medical College, Tsinghua University, Beijing, China

**Keywords:** acromegaly, adrenal insufficiency, hyperprolactinemia, hypogonadism, hypothyroidism

## Abstract

**Purpose:**

Studies on hyperprolactinemia and hypopituitarism in acromegaly are limited. We aimed to analyze the preoperative status, postoperative alterations, and correlated factors of hyperprolactinemia and hypopituitarism in acromegaly patients.

**Methods:**

This is a single-center cohort study with long-term follow-up. We prospectively enrolled 529 acromegaly patients. Hyperprolactinemia and hypopituitarism were evaluated by testing hypothalamus-pituitary-end organ (HPEO) axes hormones before and after surgery.

**Results:**

Hyperprolactinemia (39.1%) and hypopituitarism (34.8%) were common in acromegaly. The incidences of axis-specific hypopituitarism varied (hypogonadism, 29.7%; hypothyroidism, 5.9%; adrenal insufficiency, 5.1%), and multiple HPEO axes dysfunction was diagnosed in 5.3% of patients. Patients with preoperative hyperprolactinemia [hazard ratio (HR)=1.39 (1.08-1.79); *p*=0.012], hypogonadism [HR=1.32 (1.01-1.73); *p*=0.047], and hypothyroidism [HR=3.49 (1.90-6.44); *p*<0.001] had higher recurrence rates than those without. Age, sex, body mass index, tumor size, invasiveness, prolactin staining, ki-67 index, and GH/IGF-1 levels were significantly correlated with preoperative hypopituitarism and hyperprolactinemia. At median 34-month follow-up after surgery, hyperprolactinemia in 95% and axis-specific hypopituitarism in 54%-71% of patients recovered, whereas new-onset hypopituitarism (hypogonadism, 6.2%; hypothyroidism, 4.0%; adrenal insufficiency, 3.2%) was also diagnosed. A shorter tumor diameter was associated with the normalization of preoperative hyperprolactinemia after surgery. Cavernous sinus non-invasion, a shorter tumor diameter, cure at follow-up, and a lower GH nadir level were associated with the improvement of preoperative hypopituitarism after surgery. A larger tumor diameter was associated with the newly developed hypopituitarism after surgery.

**Conclusion:**

Hyperprolactinemia and hypopituitarism are common among acromegaly patients and predict worse surgical outcomes. After surgery, improvement and worsening of HPEO axes function co-exist. Correlated factors are identified for clinical management.

## Introduction

Acromegaly is a rare, slowly progressive endocrine disease with high levels of circulating growth hormone (GH) and insulin-like growth factor 1 (IGF-1) caused mostly by GH-secreting pituitary adenomas or the so-called “somatotroph adenomas” (1–3). The adenoma can cause acromegalic manifestations by producing excessive GH, hyperprolactinemia by secreting prolactin or compressing the pituitary stalk, and hypopituitarism in at least one axis by damaging the pituitary gland (4–6). Hypopituitarism in acromegaly mainly includes central hypogonadism, central hypothyroidism, and central adrenal insufficiency (1, 2, 7–9). Therefore, apart from the influence of excessive GH and IGF-1, acromegaly patients also suffer from hyperprolactinemia and hypopituitarism, causing comorbidities and worsening their quality of life and prognosis (7, 9–11).

Many studies have focused on the endocrine alterations in patients with non-functioning pituitary adenomas (NFPAs) (12–18). Evaluation for the function of the hypothalamus-pituitary-end organ (HPEO) axes was recommended for patients with macroadenomas and large microadenomas (19). However, compared with NFPA patients, the mechanism of endocrine alterations in acromegaly patients has unique features. For instance, GH can influence the gonadal function and is correlated with hypogonadism (20, 21). Mammosomatotroph adenoma or mixed somatotroph-lactotroph adenoma could directly secret prolactin and cause hyperprolactinemia (6, 22). Alleviating tumor compression on the pituitary gland and stalk, removing mammosomatotroph or mixed somatotroph-lactotroph tumors, and lowering GH and IGF-1 levels after surgery may be possible mechanisms that result in the recovery of hypopituitarism and hyperprolactinemia (6, 20, 21, 23, 24). Meanwhile, pituitary tissue also receives mechanical interference during surgery, possibly causing new-onset hypopituitarism after surgery (14, 24). Understanding the patterns of improvement or worsening of hypopituitarism and hyperprolactinemia after surgery and their correlated factors are essential in the management of acromegaly.

Unfortunately, due to the rarity of the disease, information regarding the rates and clinical correlations of hyperprolactinemia and hypopituitarism in acromegaly and the effect of surgery is limited. Previous studies were usually retrospective, enrolled small sample size, or focused only on a single axis (9, 21, 25, 26). The Pituitary Surgery Center of Neurosurgery Department at Peking Union Medical College Hospital (PUMCH) is one of the largest in China performing 800-1000 pituitary surgeries per year (27). Starting from 2015, we prospectively enrolled acromegaly patients at our institute and recorded their HPEO axes function before surgery and at follow-up. Based on the large sample size and the complete, long-term follow-up data, we aimed to address the following concerns: (i) the rate of hyperprolactinemia and the axis-specific rates of hypopituitarism among acromegaly patients and the clinicopathological correlations; (ii) whether preoperative hyperprolactinemia and hypopituitarism could predict surgical outcomes; (iii) whether and to what extent hyperprolactinemia and hypopituitarism could recover after surgery and the clinical predictors; (iv) the rates of newly-developed hyperprolactinemia and hypopituitarism after surgery and the risk factors. The results of this study would help prompt clinicians to increase their attention to the screening and management of hyperprolactinemia and hypopituitarism in acromegaly and assist patient consultation.

## Materials and Methods

### Patient Cohort and Data Collection

Acromegaly patients aged 18 and older admitted at PUMCH Neurosurgery from January 2015 to December 2018 were prospectively enrolled. The diagnosis of acromegaly was based on the Endocrine Society clinical practice guideline (28). Pregnant patients and those with polycystic ovarian syndrome, primary hypogonadism, primary hypothyroidism, and primary adrenal insufficiency were excluded. This study was carried out in accordance with the tenets of the Helsinki declaration and was approved by the Institutional Review Board at PUMCH. All patients signed the informed consent before enrollment.

Hormones of the HPEO axes were tested on each patient at baseline. Demographic information (age at diagnosis, sex, disease duration, and body mass index), tumor imaging features (diameter, cavernous sinus invasion, sphenoid sinus invasion, and optic chiasm compression), and endocrine results [fasting GH, GH nadir after the oral glucose tolerance test (OGTT), fasting IGF-1, prolactin, gonadal function, thyroid function, and adrenal function] were recorded. Hormones representing gonadal, thyroid, and adrenal functions in this study included follicle-stimulating hormone, luteinizing hormone, estrogen, progestin, testosterone, thyroid-stimulating hormone, thyroxine, triiodothyronine, free thyroxine, free triiodothyronine, adrenocorticotropic hormone, and cortisol. Disease duration was defined as the interval from the onset of acromegalic presentations to clinical diagnosis. Tumor diameter represented the widest diameter on any plane in preoperative imaging. Macroadenoma was defined as a tumor with a diameter ≥ 10 mm. On the coronal plane, a Knosp grade (29) of 3 to 4 on either side indicated tumor invasion into the cavernous sinus.

### Surgery for Pituitary Adenomas and Patient Follow-Up

All the patients received pituitary tumor resection, and all the tumors were confirmed pathologically by the histopathological and immunohistochemical (IHC) staining.

Routine postoperative follow-up was scheduled for all patients. Evaluation at follow-up included GH and IGF-1 levels, GH nadir level after OGTT, prolactin level, HPEO axes function, and pituitary imaging. Cure of acromegaly was achieved if GH<1ng/ml or GH nadir<0.4 ng/ml and IGF-1 was normalized (30). For cured patients, we recommended a follow-up frequency of every half year or one year. For patients who had a recurrence, follow-up was ceased, and repeated surgery, somatostatin analog treatment, or radiation were recommended based on individualized conditions. The follow-up time was defined as the interval from surgery to the last follow-up for cured patients or the time from surgery to recurrence for recurrent patients.

### Evaluation of Hyperprolactinemia and Hypopituitarism

The levels of HPEO axes hormones and prolactin before surgery and at follow-up were available for all patients. Serum prolactin level above the upper limit of normal confirms the diagnosis of hyperprolactinemia (31). The upper limits of normal prolactin for males and females at our institute were 15 and 25 ng/ml, respectively. Central hypogonadism, central hypothyroidism, and central adrenal insufficiency were diagnosed based on low levels of end-organ hormones (testosterone in males, estradiol in females, free thyroxine, cortisol), and low/normal levels of pituitary hormones (11, 32, 33). Patients receiving hormone replacement therapy were also identified as having hypopituitarism even though their hormones were normal. Hypofunction in two or more HPEO axes was classified as multiple HPEO axes dysfunction.

After surgery, prolactin normalization was achieved if the elevated preoperative prolactin level dropped back to normal. However, if prolactin exceeded the upper limit in the patients with normal preoperative prolactin, they were diagnosed with newly-developed hyperprolactinemia. Likewise, hypopituitarism improvement was recognized if HPEO axes hormones recovered to normal and symptoms disappeared after surgery in the patients who had preoperative hypogonadism, hypothyroidism, or adrenal insufficiency. On the contrary, if the HPEO axis function changed from normal into hypofunction, it was identified as new-onset hypopituitarism.

### Statistical Analysis

SPSS Statistics (version 26.0, IBM, USA) was used to analyze the data, and Prism (version 8.4.3, GraphPad, USA) was used to generate graphs. Categorical variables were shown as numbers and percentages. Comparisons of categorical variables were performed using the chi-squared test. Continuous variables were presented as the means ± standard deviations or medians plus interquartile range (25th and 75th percentile), according to the distribution of data evaluated by Levene’s test. Nonpaired t-tests were used to assess the differences between normally distributed continuous variables, and Mann-Whitney U tests were used with variables that failed the normality test. The false discovery rate (FDR) algorithm was adopted to control the chance of generating false positives during multiple comparisons. Statistical significance was defined as *p*<0.05. Kaplan-Meier analysis was used to analyze the probability of surgical cure among patients with or without preoperative hyperprolactinemia/hypopituitarism. A log-rank *p*<0.05 indicated a significant difference in the surgical outcomes during the postoperative follow-up.

## Results

### Clinical Features and Endocrine Alterations of Active Acromegaly Patients

Data of the 529 enrolled patients, including 235 males (44.4%) and 294 females (55.6%), were summarized in **Table 1**. Hyperprolactinemia was diagnosed in 207 patients (39.1%), and 90 (43.5%) of them had prolactin-positive adenomas. Hypopituitarism in at least one axis was found in 183 patients (34.6%). Central hypogonadism (29.7%) was the most common type of hypopituitarism, followed by central hypothyroidism (5.9%) and central adrenal insufficiency (5.1%). Twenty-eight patients (5.3%) developed multiple HPEO axes dysfunctions before surgery.

**Table 1:** Clinical and endocrine features of 529 active acromegaly patients.

	Values
Age at diagnosis, years	41.5 ± 12.2
Male, n (%)	235 (44.4%)
Body mass index, kg/m^2^	26.3 ± 3.6
Disease duration, months	60 (24, 108)
Magnetic resonance imaging features	
Tumor diameter, mm	16.7 ± 8.2
Macroadenoma, n (%)	439 (83.0%)
Cavernous sinus invasion, n (%)	175 (33.1%)
Compression of the optic chiasm, n (%)	174 (32.9%)
Sphenoid sinus invasion, n (%)	278 (52.6%)
Surgical approach and tumor pathology	
Microscopic transsphenoidal approach, n (%)	390 (73.7%)
Endoscopic transsphenoidal approach, n (%)	130 (24.6%)
Transcranial approach, n (%)	9 (1.7%)
Prolactin-positive on IHC staining, n (%)	184 (34.8%)
Ki-67 index, %	2 (1, 3)
Ki-67 index ≥ 3%, n (%)	162 (30.6%)
Endocrine hormone alterations	
Growth hormone, ng/ml	13.5 (6.9, 28.5)
Growth hormone nadir after OGTT, ng/ml	9.7 (4.5, 19.8)
Insulin-like growth factor 1, ng/ml	823 (668, 1027)
Hyperprolactinemia, n (%)	207 (39.1%)
Hypopituitarism, n (%)	183 (34.6%)
Central hypogonadism, n (%)	157 (29.7%)
Central hypothyroidism, n (%)	31 (5.9%)
Central adrenal insufficiency, n (%)	27 (5.1%)
Multiple HPEO axes dysfunctions, n (%)	28 (5.3%)

Continuous variables are presented as means ± standard deviations if normally distributed or medians (25th and 75th quartile) if not normally distributed.

HPEO, hypothalamic-pituitary-end organ; IHC, immunohistochemical; OGTT, oral glucose tolerance test.

### Clinicopathological Correlations of Hyperprolactinemia and Hypopituitarism

Patients with hyperprolactinemia were younger (*p*=0.006) and more likely to be females (*p*=0.001), had larger and more invasive pituitary adenomas (*p*<0.001), and had higher levels of GH (*p*=0.004) and GH nadir (*p*=0.003), compared to patients with normal prolactin (**Table 2**).

**Table 2:** Clinicopathological correlations of hyperprolactinemia and hypopituitarism in acromegaly.

	Hyperprolactinemia				Central hypogonadism				Central hypothyroidism				Central adrenal insufficiency		
	Yes (n = 207)	No (n = 322)	*p*		Yes (n = 157)	No (n = 372)	*p*		Yes (n = 31)	No (n = 498)	*p*		Yes (n = 27)	No (n = 502)	*p*
Age at diagnosis, years	39.6 ± 11.8	42.6 ± 12.3	0.006	^#^	38.1 ± 11.2	42.9 ± 12.3	<0.001	^#^	41.3 ± 14.0	41.5 ± 12.1	0.950		37.2 ± 12.1	41.7 ± 12.2	0.064
Male, n (%)	74 (35.7%)	161 (50.0%)	0.001	^#^	89 (56.7%)	146 (39.2%)	<0.001	^#^	7 (22.6%)	228 (45.8%)	0.012	^#^	16 (59.3%)	219 (43.6%)	0.111
Body mass index, kg/m^2^	26.2 ± 3.5	26.4 ± 3.7	0.625		27.1 ± 3.7	26.0 ± 3.5	0.001	^#^	24.5 ± 3.3	26.4 ± 3.6	0.004	^#^	25.2 ± 3.8	26.4 ± 3.6	0.099
Disease duration, months	60 (24, 108)	60 (32, 108)	0.173		60 (30, 108)	60 (24, 120)	0.520		60 (36, 120)	60 (24, 108)	0.466		60 (42, 90)	60 (24, 108)	0.662
Tumor diameter, mm	19.1 ± 8.6	15.1 ± 7.5	<0.001	^#^	20.6 ± 9.0	15.1 ± 7.1	<0.001	^#^	21.7 ± 8.7	16.4 ± 8.0	<0.001	^#^	18.9 ± 8.8	16.6 ± 8.1	0.153
Macroadenoma, n (%)	187 (90.3%)	252 (78.3%)	<0.001	^#^	145 (92.4%)	294 (79.0%)	<0.001	^#^	30 (96.8%)	409 (82.1%)	0.035		26 (96.3%)	413 (82.3%)	0.104
Cavernous sinus invasion, n (%)	96 (46.4%)	79 (24.5%)	<0.001	^#^	75 (47.8%)	100 (26.9%)	<0.001	^#^	21 (67.7%)	154 (30.9%)	<0.001	^#^	12 (44.4%)	163 (32.5%)	0.198
Optic chiasm compression, n (%)	89 (43.0%)	85 (26.4%)	<0.001	^#^	71 (45.2%)	103 (27.7%)	<0.001	^#^	20 (64.5%)	154 (30.9%)	<0.001	^#^	11 (40.7%)	163 (32.5%)	0.373
Sphenoid sinus invasion, n (%)	135 (65.2%)	143 (44.4%)	<0.001	^#^	112 (71.3%)	166 (44.6%)	<0.001	^#^	22 (71.0%)	256 (51.4%)	0.034		17 (63.0%)	261 (52.0%)	0.266
Prolactin+ on IHC staining, n (%)	90 (43.5%)	94 (29.2%)	0.001	^#^	43 (27.4%)	141 (37.9%)	0.020	^#^	7 (22.6%)	177 (35.5%)	0.142		9, (33.3%)	175 (34.9%)	0.871
Tumor Ki-67 index value, n	2 (1, 3)	2 (1, 3)	0.162		2 (1, 3)	2 (1, 3)	0.007	^#^	2 (1, 3)	2 (1, 3)	0.297		2 (2, 3)	2 (1, 3)	0.189
Tumor Ki-67 index ≥ 3%, n (%)	70 (33.8%)	92 (28.6%)	0.201		62 (39.5%)	100 (26.9%)	0.004	^#^	15 (48.4%)	147 (29.5%)	0.027		12 (44.4%)	150 (29.9%)	0.110
Growth hormone, ng/ml	16.3 (9.2, 34.1)	11.2 (5.9, 23.1)	0.004	^#^	22.4 (8.4, 61.2)	11.6 (6.5, 21.8)	<0.001	^#^	13.2 (8.1, 35.8)	13.6 (6.8, 28.2)	0.782		32.1 (9.0, 62.3)	13.3 (6.8, 26.7)	0.206
Growth hormone nadir, ng/ml	12.0 (2.1, 63.3)	7.9 (3.9, 17.7)	0.003	^#^	15.3 (6.3, 33.4)	8.3 (4.1, 16.1)	0.001	^#^	9.1 (4.8, 28.2)	9.7 (4.5, 19.6)	0.910		19.4 (6.6, 44.1)	9.4 (4.5, 19.2)	0.212
Insulin-like growth factor 1, ng/ml	846 (677, 1068)	808 (667, 985)	0.090		875 (717, 1087)	799 (659, 988)	0.007	^#^	680 (484, 797)	831 (677, 1037)	<0.001	^#^	790 (665, 1073)	824 (668, 1021)	0.897

Continuous variables are presented as means ± standard deviations if normally distributed or medians (25th and 75th quartile) if not normally distributed.

IHC, immunohistochemical; OGTT, oral glucose tolerance test

^#^Indicates statistically significant differences after being justified by the FDR algorithm, used to control the chance of generating false positives during multiple comparisons.

Among those with hyperprolactinemia (n=207), patients with prolactin-negative adenomas (n=117) had larger tumors (*p*=0.003), and their tumors were more likely to invade into cavernous sinus (*p*=0.004) and sphenoidal sinus (*p*=0.003), compared to the patients with prolactin-positive adenomas (n=90).

Among all the patients with prolactin-positive tumors (n=184), those with hyperprolactinemia (n=90) had larger tumors (*p*<0.001) and higher levels of GH (*p*=0.026) and GH nadir (*p*=0.024), and their tumors were more likely to invade into cavernous sinus (*p*=0.003) and sphenoidal sinus (*p*=0.001), compared to the patients without hyperprolactinemia (n=94).

Patients with hypogonadism were younger (*p*<0.001), fatter (*p*=0.001), and more likely to be males (*p*<0.001), had larger and more invasive tumors (*p*<0.001) and greater ki-67 index (*p*<0.01), and had higher levels of GH (*p*<0.001), GH nadir (*p*=0.001) and IGF-1 (*p*=0.007), compared to patients with normal gonadal function. 74 of patients (47.1%) with hypogonadism had hyperprolactinemia, while only 35.8% of patients with hypogonadism had hyperprolactinemia (*p*=0.014). Patients with hypothyroidism were thinner (p=0.004) and more likely to be females (*p*=0.012), had larger and more invasive tumors, and lower levels of IGF-1 (*p*<0.001), compared to patients with normal thyroid function. No differences were found regarding clinicopathological characteristics between patients with and without adrenal insufficiency or multiple HPEO axes dysfunctions.

### Preoperative Hyperprolactinemia and Hypopituitarism Predict Worse Surgical Outcomes

**Figure 1:**
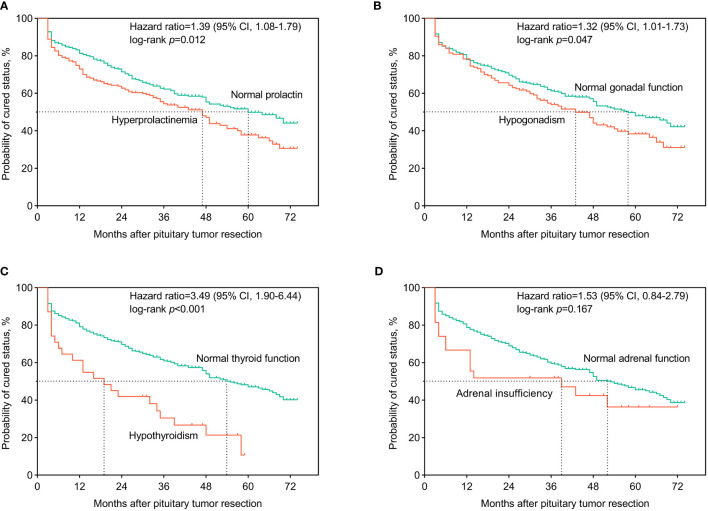
Kaplan-Meier curves for surgical outcomes among acromegaly patients. The patients were classified according to whether or not having developed preoperative hyperprolactinemia **(A)**, central hypogonadism **(B)**, central hypothyroidism **(C)**, and central adrenal insufficiency **(D)**. Hazard ratio and 95% credibility interval (95% CI) of hyperprolactinemia and axis-specific hypopituitarism on the surgical outcomes, compared to normal hypothalamus-pituitary-end organ axes functions, were calculated and recorded on the panels. Log-rank tests were used to test the differences in the recurrence/cure rates after surgery between groups.

In **Figure 1**, we present the Kaplan-Meier curves for follow-up among patients with acromegaly. Patients with preoperative hyperprolactinemia had a higher recurrence rate and shorter median recurrence time after surgery than those with normal prolactin (HR, 1.39 (1.08-1.79); log-rank *p*=0.012]. Patients with preoperative hypogonadism had a higher recurrence rate and shorter median recurrence time than those with normal gonadal function (HR, 1.32 (1.01-1.73); log-rank *p*=0.047]. Patients with preoperative hypothyroidism had a higher recurrence rate and shorter median recurrence time than those with normal thyroid function [HR, 3.49 (1.90-6.44); log-rank *p*<0.001].

### Effect of Pituitary Tumor Resection on Endocrine Hormones in Acromegaly

**Figure 2:**
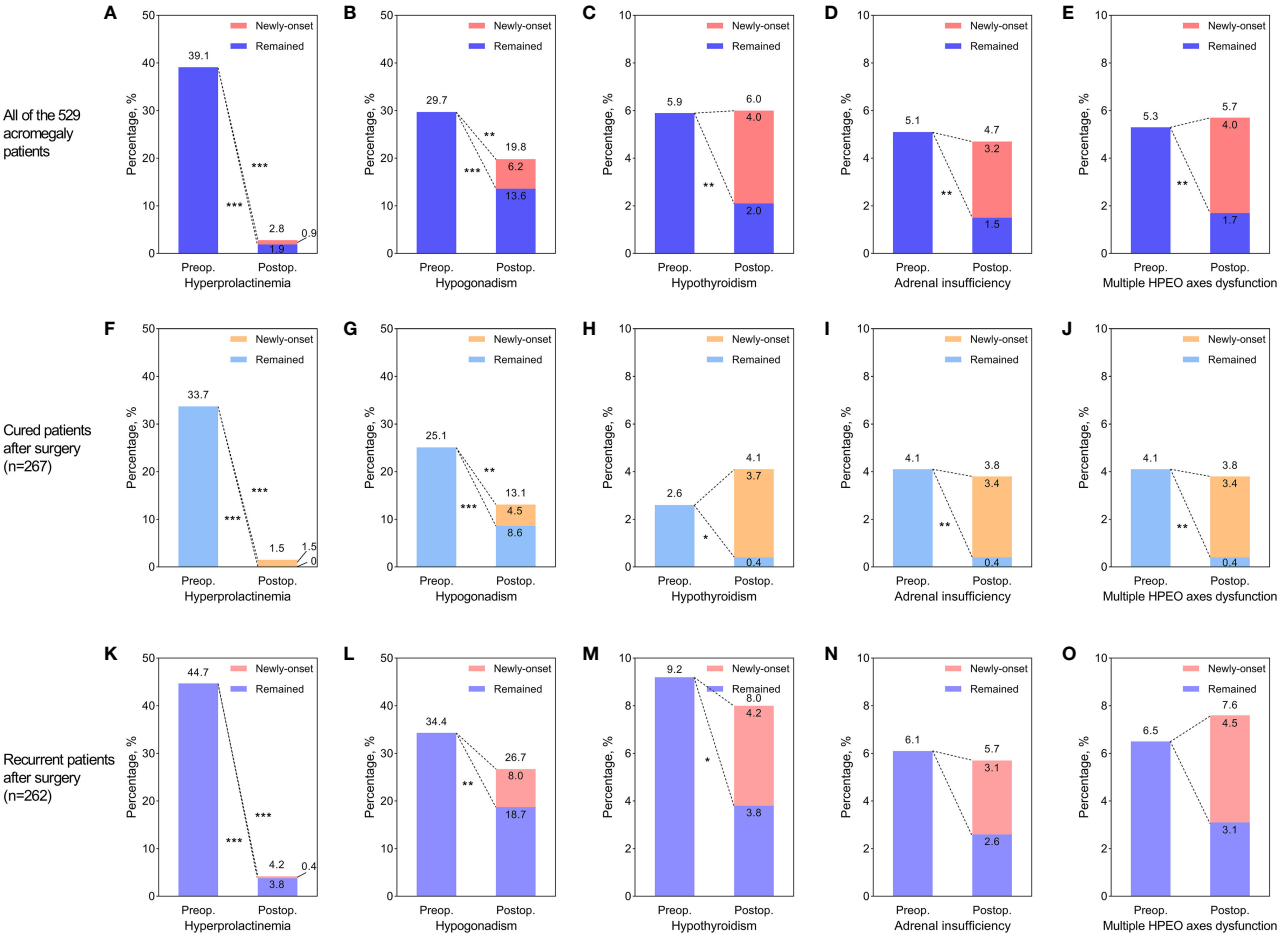
The effect of surgery on hyperprolactinemia and hypopituitarism in all acromegaly patients **(A–E)**, cured patients **(F–J)**, and recurrent patients **(K–O)**. **(A, F, K)** Improvement of preoperative hyperprolactinemia and newly-developed postoperative hyperprolactinemia. **(B, G, L)** Improvement of preoperative hypogonadism and newly-developed postoperative hypogonadism. **(C, H, M)** Improvement of preoperative hypothyroidism and newly-developed postoperative hypothyroidism. **(D, I, N)** Improvement of preoperative adrenal insufficiency and newly-developed postoperative adrenal insufficiency. **(E, J, O)** Improvement of preoperative multiple hypothalamus-pituitary-end organ (HPEO) axes dysfunctions and newly-developed postoperative multiple HPEO axes dysfunctions. The stars to the lower left of the dotted lines indicated that the improvement of hyperprolactinemia or hypopituitarism after surgery was significant. The stars to the upper right of the dotted lines indicated that the differences between preoperative rates of hyperprolactinemia/hypopituitarism and postoperative rates were significant. * indicated *p*<0.05, ** indicated *p*<0.01, and *** indicated *p*<0.001. Preop, preoperative; Postop, postoperative.

The median follow-up time after surgery was 34 (16, 53) months. At the latest follow-up, 267 patients (50.5%) were in endocrine remission, while 262 (49.5%) experienced recurrence. While the prolactin levels in most patients with hyperprolactinemia returned to normal after surgery, hyperprolactinemia remained in 10 patients (1.9%). Six of these ten patients had prolactin-positive adenomas, and all of them experienced recurrence during follow-up. Additionally, five patients (0.9%) developed new-onset hyperprolactinemia, and the rate of postoperative hyperprolactinemia rose to 2.8%. The gonadal function in about half of the patients with preoperative hypogonadism recovered after surgery. With 33 patients (6.2%) developing new-onset postoperative hypogonadism, the rate of hypogonadism at follow-up was 19.8%. The thyroid function in two-thirds of the patients with preoperative hypothyroidism (5.9% of all) recovered after surgery, while 2.0% of patients didn’t. Twenty-one patients (4%) developed new-onset postoperative hypothyroidism, and the hypothyroidism rate at follow-up was 6.0%. More than 70% of the preoperative adrenal insufficiency (5.1% of all) recovered after surgery, while 17 patients (3.2%) developed newly-onset postoperative adrenal insufficiency. Thus, the rate of adrenal insufficiency at follow-up was 4.7%. Of the 28 patients diagnosed with multiple HPEO axes dysfunction (5.3% of all) before surgery, 68% recovered at follow-up. Unfortunately, 21 patients (4%) developed multiple HPEO axes dysfunction after surgery, and the rate at follow-up turned up to 5.7%. Therefore, as shown in **Figure 2**, the preoperative hyperprolactinemia (*p*<0.001), hypogonadism (*p*<0.001), hypothyroidism (*p*=0.002), adrenal insufficiency (*p*=0.002), and multiple HPEO axes dysfunctions (*p*=0.002) all improved significantly after surgery, especially in the cured patients. However, adrenal insufficiency and multiple HPEO axes dysfunctions in the recurrent patients did not remarkably improve. Given that some patients developed new-onset hyperprolactinemia and hypopituitarism after surgery, only the rates of hyperprolactinemia (39.1% to 2.8%, *p*<0.001) and hypogonadism (29.7% to 19.8%, *p*=0.004) decreased at follow-up. In subgroups, the rates of hyperprolactinemia and hypogonadism decreased in cured patients, while only the rate of hyperprolactinemia decreased in recurrent patients.

### Predictors of Prolactin Normalization and Hypopituitarism Improvement and Risk Factors of Newly Developed Hypopituitarism After Surgery

In **Table 3**, we present that a shorter tumor maximal diameter (*p*=0.001) was associated with the normalization of preoperative hyperprolactinemia after surgery. Cavernous sinus non-invasion (*p*=0.001), a shorter tumor diameter (*p*=0.001), cure at follow-up (*p*=0.002), and a lower GH nadir level (*p*=0.009) were associated with the improvement of preoperative hypopituitarism after surgery.

**Table 3:** Predictors of prolactin normalization and hypopituitarism improvement after surgery.

	Normalization of Preoperative Hyperprolactinemia				Improvement of Preoperative Hypopituitarism			
	Yes (n=197)	No (n=10)	*p*		Yes (n=98)	No (n=85)	*p*	
Preoperative index								
Age at diagnosis, years	39.5 ± 11.9	43.0 ± 9.3	0.358		38.0 ± 10.1	38.4 ± 13.0	0.821	
Male, n (%)	68 (34.5%)	6 (60.0%)	0.193		56 (57.1%)	37 (43.5%)	0.066	
Body mass index, kg/m^2^	26.1 ± 3.5	28.3 ± 3.1	0.053		26.1 ± 3.2	27.3 ± 4.3	0.026	
Disease duration, months	60 (24, 96)	96 (45, 120)	0.358		60 (31.5, 93)	60 (36, 120)	0.201	
Tumor diameter, mm	18.7 ± 8.2	27.5 ± 11.7	0.001	#	18.3 ± 8.0	22.9 ± 9.9	0.001	#
Cavernous sinus invasion, n (%)	88 (44.7%)	8 (80.0%)	0.063		37 (37.8%)	53 (62.4%)	0.001	#
Optic chiasm compression, n (%)	85 (43.1%)	4 (40.0%)	1.000		40 (40.8%)	44 (51.8%)	0.138	
Sphenoid sinus invasion, n (%)	125 (63.5%)	10 (100%)	0.043		62 (63.3%)	65 (76.5%)	0.053	
Growth hormone, ng/ml	15.9 (9.2, 33.8)	29.3 (12.9, 60.2)	0.392		21.9 (9.9, 59.0)	15.8 (7.5, 59.1)	0.201	
Growth hormone nadir, ng/ml	11.9 (6.0, 22.6)	19.1 (7.9, 33.9)	0.395		18.0 (8.0, 30.3)	9.9 (4.3, 31.5)	0.232	
Insulin-like growth factor 1, ng/ml	870.5 ± 281.2	997.2 ± 379.5	0.173		906.1 ± 280.1	850.1 ± 302.8	0.196	
Postoperative index								
MTS approach, n (%)	140 (71.1%)	10 (100%)	0.102		75 (76.5%)	62 (72.9%)	0.577	
ETS approach, n (%)	53 (26.9%)	0 (0%)	0.126		22 (22.4%)	17 (20.0%)	0.687	
Cure at follow-up, n (%)	90 (45.7%)	0 (0%)	0.012		50 (51.0%)	24 (28.2%)	0.002	#
Prolactin+ on IHC staining, n (%)	84 (42.6%)	6 (60.0%)	0.451		31 (31.6%)	19 (22.4%)	0.160	
Tumor Ki-67 index value, n	2 (1, 3)	2 (2, 3)	0.880		2 (2, 3)	2 (1, 3)	0.589	
Follow-up time, months	35 (12, 51)	8.5 (3.8, 33.3)	0.035		38.5 (16, 57)	25 (11, 44)	0.020	
Growth hormone, ng/ml	2.0 (0.7, 4.0)	7.0 (3.3, 24.7)	0.055		1.7 (0.4, 4.3)	2.2 (0.9, 6.1)	0.029	
Growth hormone nadir, ng/ml	0.9 (0.3, 2.4)	4.1 (1.8, 15.9)	0.288		0.7 (0.2, 2.7)	1.8 (0.7, 6.7)	0.009	#
Insulin-like growth factor 1, ng/ml	382.7 ± 232.0	678.3 ± 394.8	0.043		388.6 ± 235.8	436.0 ± 316.1	0.257	

Continuous variables are presented as means ± standard deviations if normally distributed or medians (25th and 75th quartile) if not normally distributed.

ETS, endoscopic transsphenoidal; IHC, immunohistochemical; MTS, microscopic transsphenoidal; OGTT, oral glucose tolerance test.

^#^Indicates statistically significant differences after being justified by the FDR algorithm.

In **Table 4**, we present that patients with a larger tumor (*p*=0.002) were more likely to develop hypopituitarism after surgery.

**Table 4:** Risk factors of newly-developed hyperprolactinemia and hypopituitarism after surgery.

	Newly-Developed Hyperprolactinemia				Newly-Developed Hypopituitarism			
	Yes (n=5)	No (n=317)	*p*		Yes (n=38)	No (n=308)	*p*	
Preoperative index								
Age at diagnosis, years	37.2 ± 14.0	42.7 ± 12.3	0.321		40.5 ± 11.6	43.5 ± 12.2	0.139	
Male, n (%)	0 (0%)	161 (50.8%)	0.071		10 (26.3%)	132 (42.9%)	0.050	
Body mass index, kg/m^2^	25.1 ± 4.0	26.4 ± 3.7	0.425		26.7 ± 3.4	26.0 ± 3.5	0.304	
Disease duration, months	24 (12, 36)	60 (36, 108)	0.082		36 (24, 99)	60 (24, 120)	0.319	
Tumor diameter, mm	11.8 ± 4.7	15.2 ± 7.5	0.314		17.6 ± 7.7	14.4 ± 6.6	0.002	#
Cavernous sinus invasion, n (%)	1 (20.0%)	78 (24.6%)	1.000		16 (42.1%)	69 (22.4%)	0.008	
Optic chiasm compression, n (%)	0 (0%)	85 (26.8%)	0.402		16 (42.1%)	74 (24.0%)	0.017	
Sphenoid sinus invasion, n (%)	1 (20.0%)	142 (44.8%)	0.513		21 (55.3%)	130 (42.2%)	0.126	
Growth hormone, ng/ml	4.5 (3.9, 6.0)	11.3 (5.9, 23.8)	0.307		12.3 (6.8, 28.9)	11.5 (6.3, 20.9)	0.203	
Growth hormone nadir, ng/ml	3.6 (3.3, 3.9)	8.0 (4.0, 17.9)	0.355		10.0 (4.0, 20.4)	8.2 (4.0, 15.3)	0.183	
Insulin-like growth factor 1, ng/ml	750.2 ± 108.3	836.5 ± 253.1	0.307		811.9 ± 234.5	839.2 ± 253.6	0.529	
Postoperative index								
MTS approach, n (%)	3 (60.0%)	237 (74.8%)	0.815		24 (63.2%)	229 (74.4%)	0.142	
ETS approach, n (%)	2 (40.0%)	75 (23.7%)	0.748		13 (34.2%)	78 (25.3%)	0.240	
Cure at follow-up, n (%)	4 (80.0%)	173 (54.6%)	0.496		13 (34.2%)	174 (56.5%)	0.009	
Prolactin+ on IHC staining, n (%)	2 (40.0%)	92 (29.0%)	0.968		15 (39.5%)	119 (38.6%)	0.920	
Tumor Ki-67 index value, n	3 (2, 5)	2 (1, 3)	0.123		2 (1, 3)	2 (1, 3)	0.241	
Follow-up time, months	46.8 ± 13.1	36.2 ± 21.1	0.263		31.9 ± 21.2	36.7 ± 21.3	0.193	
Growth hormone, ng/ml	0.9 (0.4, 1.0)	1.3 (0.4, 3.0)	0.693		1.8 (0.6, 3.7)	1.4 (0.4, 2.9)	0.974	
Growth hormone nadir, ng/ml	0.4 (0.3, 0.9)	0.5 (0.2, 2.1)	0.551		0.6 (0.3, 2.3)	0.5 (0.2, 1.5)	0.219	
Insulin-like growth factor 1, ng/ml	216 (216, 219)	262.0 (187.8, 421.8)	0.315		286 (212, 433.3)	272 (190.8, 413.3)	0.549	

Continuous variables are presented as means ± standard deviations if normally distributed or medians (25th and 75th quartile) if not normally distributed.

ETS, endoscopic transsphenoidal; IHC, immunohistochemical; MTS, microscopic transsphenoidal; OGTT, oral glucose tolerance test.

^#^Indicates statistically significant differences after being justified by the FDR algorithm.

## Discussion

The current study systematically analyzed the preoperative status, postoperative alterations, and correlated clinicopathological factors of hyperprolactinemia and hypopituitarism in 529 acromegaly patients who were prospectively enrolled and followed up in a single center. The results showed that nearly two-fifths of acromegaly patients developed hyperprolactinemia before surgery, and over one-third had hypopituitarism in at least one axis. The most common single-axis hypopituitarism was hypogonadism, followed by hypothyroidism and adrenal insufficiency. Multiple HPEO axes dysfunction was diagnosed in over 5% of acromegaly patients. Patients with preoperative hyperprolactinemia, hypogonadism, or hypothyroidism were more likely to experience tumor recurrence after surgery than those without. Hyperprolactinemia in 95% of patients and hypopituitarism in over half of patients recovered after surgery. In addition, newly-developed hypogonadism, hypothyroidism, and adrenal insufficiency occurred in ~5% of patients at follow-up, in both cured and recurrent patients. Factors correlated with preoperative hyperprolactinemia and hypopituitarism and the predictors for their improvement and worsening after surgery were analyzed.

The rate of hyperprolactinemia in the acromegaly population was reported to range from 25% to nearly one-third (3, 6, 8). Our result showed that the rate was 39.1%, and the female to male ratio was 2:1. 43.5% of patients with hyperprolactinemia had prolactin-positive tumors, suggesting that prolactin hypersecretion by tumor might be a primary etiology in these patients. Arafah and colleagues (13) proposed that high intrasellar pressure was a mechanism for hyperprolactinemia. Our result paralleled this hypothesis and showed that macroadenomas and sinus invasion were correlated with hyperprolactinemia in patients with prolactin-negative tumors compared to those with prolactin-positive tumors, implying that prolactin-negative adenomas could cause hyperprolactinemia through compressing the pituitary stalk and elevating intrasellar pressure. After surgery, hyperprolactinemia recovered in most patients. Whether the patients were cured or not had little impact on the recovery. We also found that preoperative hyperprolactinemia was more likely to recover in patients who had smaller pituitary adenomas.

Whether preoperative hypopituitarism and hyperprolactinemia could predict the surgical outcome is unclear. Our data revealed that patients with hyperprolactinemia, hypogonadism, and hypothyroidism had higher recurrence rates after surgery than those without. Therefore, evaluation of HPEO axes functions is needed among acromegaly patients because the result could provide us with clues whether we should conduct more intensive follow-up and subsequent in-time intervention for this group of patients.

Studies showed the rates of HPEO axes dysfunctions varied by axis and generally presented in the following sequence: GH deficiency, hypogonadism, hypothyroidism, and adrenal insufficiency (17, 23). The rates of hypopituitarism in the current study were in the same order. After surgery, improvement and worsening of HPEO axes dysfunctions co-existed, and the rate of hypopituitarism recovery was higher than the incidence of new-onset hypopituitarism (14). Previous studies revealed that 37.4% to 57% of patients with preoperative hypopituitarism showed normalized function after surgery (12, 15, 16, 18, 24), whereas newly-developed HPEO axes dysfunction was found in 10% to 30% of patients (1, 12, 18).

Hypogonadism is the most prevalent type of single-axis hypopituitarism caused by mass effect, GH excess, or hyperprolactinemia (20, 21). Of the 363 patients in Katznelson’s study, 53% were diagnosed with hypogonadism (20). However, only 30% of the 529 patients in our cohort presented with hypogonadism. One reason might be the different definitions of hypogonadism. In Katznelson’s study, all the female patients with amenorrhea who had no history of surgical menopause and all the male patients with only a single value of low serum testosterone were diagnosed with hypogonadism, which probably expanded the sample size by enrolling unexpected patients, e.g. primary amenorrhea, or error/variation in hormone test (10, 20). Grynberg and colleagues (21) found that hypogonadism was common among women of reproductive age, and we also confirmed that young age was correlated with hypogonadism. Central hypogonadism occurred more commonly in patients with adenomas of wider diameter, greater ki-67 index, and higher invasiveness. Since high intrasellar pressure was also proposed as a major mechanism for hypopituitarism (13), all factors that could increase the intrasellar pressure could prompt the pathogenesis of hypopituitarism. In addition, GH and IGF-1 levels also influence hypothalamic-pituitary-gonadal axis hormones. Patients with high levels of GH, GH nadir, and IGF-1 were more likely to develop hypogonadism, with GH/IGF-1 inhibition on the hypothalamic-pituitary-gonadal axis being a possible etiology (20, 21). Hypogonadism could recover in two-thirds of acromegaly patients after surgery (34, 35). In this study, the rate was 54.2% and was higher in cured patients.

A recent study showed that the incidence of central hypothyroidism was 8.7% among acromegaly patients, far lower than that in NFPA patients, which might result from the stimulation effect of GH/IGF-1 on thyroid function (9). Our data revealed that 5.9% of acromegaly patients had hypothyroidism, and the IGF-1 levels in these patients were remarkably lower than those with normal thyroid function. After surgery, hypothyroidism could recover in both cured and recurrent patients.

Adrenal insufficiency is the most severe type of endocrine deficit, and increases mortality and impaired quality of life in the long term (11). In this cohort, 5% of patients had central adrenal insufficiency at baseline. One Dutch study showed that the rate of adrenal insufficiency one year after surgery was low (9%), and the late-onset adrenal insufficiency is infrequent in cured patients (25). Our follow-up data characterized that 90% of preoperative central adrenal insufficiency in the cured acromegaly patients recovered after surgery. However, for all the patients as a whole, although 3.6% had their adrenal insufficiency recovered, 3.2% of patients developed new-onset adrenal insufficiency, leading to a postoperative rate of 4.7%, similar to the preoperative level.

The current study has some limitations. First, the patients were enrolled from only one medical center, leading to possible selection bias compared with multi-center research. However, the Pituitary Surgery Center at PUMCH Neurosurgery is the founder of China Pituitary Adenoma Specialist Council and China Pituitary Disease Registry Center. Acromegaly patients from all over the country come to our institute for treatment. By reviewing our database, we found that the patients were from 30 of 34 provinces in China, revealing a fair representation of the overall Chinese population. Second, the median follow-up time was only 34 months. A longer follow-up is still needed. Third, although our sample size of 529 acromegaly patients was relatively large, the numbers of patients in some subgroups were not enough to reach statistical significance given the low incidence of hypothyroidism and adrenal insufficiency in acromegaly. A larger sample size might be a potential means to avoid false-negative results and to verify the result from this analysis.

## Conclusions

More than one-third of acromegaly patients suffer from hyperprolactinemia or hypopituitarism. Central hypogonadism is the most common hypopituitarism in acromegaly. Preoperative hyperprolactinemia, hypogonadism, and hypothyroidism can predict worse surgical outcomes. After surgery, hyperprolactinemia and HPEO axes dysfunction are recoverable, especially in cured patients. However, approximately 5% of patients develop new-onset hyperprolactinemia or hypopituitarism. Tumor diameter, cavernous sinus invasion status, and the remission state and GH nadir levels at follow-up are key factors predicting the recovery or worsening of hyperprolactinemia and hypopituitarism after surgery. This study with the largest sample size and intact follow-up data provides deep insight into the preoperative status of hyperprolactinemia and axis-specific hypopituitarism in acromegaly and the effect of surgery and clinical correlations.

## Data Availability Statement

The raw data supporting the conclusions of this article will be made available by the authors, without undue reservation.

## Ethics Statement

The studies involving human participants were reviewed and approved by the institutional review board at Peking Union Medical College Hospital, Chinese Academy of Medical Sciences and Peking Union Medical College. The patients/participants provided their written informed consent to participate in this study.

## Author Contributions

XG, RZ, DZ, and BX designed the study. XG, ZW, LG, YoY, KD, XB, MF, ZX, YiY, WL, RW, WM, and BX enrolled patients, provided clinical care, and completed follow-up. XG and BX monitored the entire procedure and made quality control of the study. XG, RZ, and DZ collected data and performed statistical analysis and visualization of the data. XG wrote the manuscript, and RZ, DZ, and BX revised the manuscript. The whole team approved the final version of the manuscript.

## Conflict of Interest

The authors declare that the research was conducted in the absence of any commercial or financial relationships that could be construed as a potential conflict of interest.

## Publisher’s Note

All claims expressed in this article are solely those of the authors and do not necessarily represent those of their affiliated organizations, or those of the publisher, the editors and the reviewers. Any product that may be evaluated in this article, or claim that may be made by its manufacturer, is not guaranteed or endorsed by the publisher.

## Abbreviations

FDR, false discovery rate; GH, growth hormone; HPEO, hypothalamus-pituitary-end organ; HR, hazard ratio; IGF-1, insulin-like growth factor 1; IHC, immunohistochemical; NFPA, non-functioning pituitary adenoma; OGTT, oral glucose tolerance test; PUMCH, Peking Union Medical College Hospital.
